# Developmental dyslexia in children with perinatal exposure to hypoxia: A systematic review

**DOI:** 10.1371/journal.pone.0308497

**Published:** 2024-09-12

**Authors:** Bartosz M. Radtke, Małgorzata Lipowska, Łucja Bieleninik, Ariadna Łada-Maśko, Katarzyna Krempla-Patron, Rafał Nowicki, Gabriela Gradys, Anna Brykała, Judyta Pacuła, Marek Arasimowicz, Urszula Sajewicz-Radtke

**Affiliations:** 1 Laboratory of Psychological and Educational Tests, Gdańsk, Poland; 2 Institute of Psychology, University of Gdansk, Gdańsk, Poland; 3 Institute of Pedagogy and Languages, University of Applied Sciences in Elbląg, Elbląg, Poland; 4 The Grieg Academy Music Therapy Research Centre, NORCE Norwegian Research Centre, Bergen, Norway; 5 Neurologopedic Therapy Center, Gdańsk, Poland; University of Padova, ITALY

## Abstract

**Background:**

Research on a health-related event at the stage of labour and the occurrence of adverse outcomes at the school age has provided inconclusive results. Thus far, no systematic reviews have been conducted. Thus, the objective of this study is to investigate the association between hypoxia during pregnancy or delivery and the subsequent occurrence of a developmental dyslexia in school-age children.

**Methods:**

We used a standard search strategy of electronic databases (PubMed, PsycINFO, Web of Science, EMBASE, and Cochrane Library) and handsearching. We included observational studies (cohort studies, case-control studies) that consider as an exposure the presence of hypoxia during pregnancy or delivery, and as an outcome, developmental dyslexia in school-age children. Two reviewers independently conducted the search and determined eligibility, which was not restricted by language or year of publication.

**Results:**

The search took place until 1 April 2023. Of the 1,336 abstracts screened, 6 were assessed for eligibility. Of the six eligible studies, no studies met the pre-specified eligibly criteria.

**Conclusions:**

We were unable to assess the association between hypoxia and developmental dyslexia, as no eligible studies were found. Thus, the association between hypoxia during pregnancy or delivery and dyslexia in school-age children remains unknown.

## Introduction

The latest edition of the International Statistical Classification of Diseases and Related Health Problems (International Classification of Diseases) by the World Health Organization [1] introduced a new term "developmental learning disorder" instead of the current one: "specific developmental disorders of scholastic skills" [2]. This category of disorders includes, among others: developmental learning disorder with impairment in reading, commonly referred to as dyslexia [1]. Developmental dyslexia is a neurodevelopmental disorder identified in children with average intelligence and sensory capabilities. It is characterised by difficulties in reading including in accurate and/or fluid word recognition, spelling, and decoding skills across different languages. Dyslexia is considered a neurodevelopmental disorder, because it arises from the natural neurological variations in brain development, which deviate from the standard developmental path [3]. Ramus et al. [4] emphasise that some cases of dyslexia (a minority) might be explained by non-phonological deficits such as in the automaticity hypothesis [5] or visual theories [6–8].

However, since there are at least two empirically documented trends in the search for the causes of dyslexia (leading phonological and niche non-phonological), it should be recognised as a disorder stemming from various causes. Reading is a highly complex, multimodal cognitive skill. The act of reading must involve the coordinated action of many areas of the brain involved in both lower and higher order processing. Neuroimaging research has delineated a set of brain systems specialised in various sub-skills involved in reading that form a "reading network" [9, 10]. A problem in the operation or interaction of one or more parts of these systems seriously hampers the acquisition of fluent reading by people with dyslexia [11]. Thus, this complex network of systems may be susceptible to the disturbing influence of many factors throughout brain development, both in the pre- and perinatal period.

Furthermore, it has been established that genetics play a significant role in causing dyslexia [12–14]. However, a study on environmental factors focusing on inadequate oxygen supply during pregnancy or labour also presents an intriguing area of research for uncovering the pathomechanism of dyslexia [15]. Most perinatal injuries arise from hypoxia, which is defined as inadequate oxygenation of body tissues, and the conditions associated with it [16]. Various medical conditions can contribute to asphyxia. These conditions may lead to different types of hypoxic events (prenatal or neonatal, chronic or acute) and also to different outcomes [17, 18]. The diagnostic criteria for neonatal hypoxia are based on a set of markers including Apgar below 5 (at the fifth minute), need for intubation or cardiopulmonary resuscitation (CPR), umbilical artery pH below 7.00, and abnormal neurological signs such as hypotonic muscles or an absent sucking reflex [17]. Among impacted infants, a quarter experience profound and enduring neuropsychological consequences [19], encompassing conditions such as cognitive impairment, visual motor or visual perception issues, increased hyperactivity, cerebral palsy, and epilepsy [20, 21]. Hypoxic-ischemic encephalopathy (HIE) is a cerebral injury resulting from oxygen deprivation due to either hypoxic or anoxic incidents [22, 23]. Hypoxia signifies a reduction in blood oxygen reaching the brain, while ischemia denotes a decreased blood flow to the brain. The prevalence of HIE is estimated at 1–8 cases per 1,000 live full-term births [20, 24]. Some studies highlight that some patients with HIE may develop learning disabilities [21–23, 25–27]. Of course, the severity of neurological consequences depends not only on the severity of HIE but also on the therapeutic interventions employed. One of the most well-researched therapeutic methods is therapeutic hypothermia. Clinical studies have established that therapeutic cooling improves neurological outcomes following various acute brain injuries, including neonatal hypoxia-ischemia [28–30].

There are also studies indicating the relationship between smoking by pregnant mothers and reading difficulties in their school-age children [31–33] and it has been proven that one of the causes of fetal hypoxia is smoking by the mother during pregnancy [34, 35].

For example, a study conducted in China [15] on complicated natural childbirth and perinatal hypoxia confirmed a higher occurrence of dyslexic children in these populations compared to those without dyslexia (3.12% vs. 1.75% and 3.76% vs. 1.42% of the overall sample for difficult natural delivery and perinatal hypoxia, respectively).

### Previous research and rationale for the review

As per our current knowledge, no review or meta-analysis has systematically examined the link between exposure to prenatal/perinatal hypoxia and the emergence of developmental dyslexia symptoms during the school-age period. This absence prompted us to undertake the current systematic review of previously conducted observational studies including those of both cohort and case-control designs. These studies investigate the presence of hypoxia during pregnancy or delivery as the exposure factor and its correlation with dyslexia in children of school age, as defined by the ICD-11 classification of neurodevelopmental disorders. By delving into the association between the health-related event, i.e., hypoxia during pregnancy or delivery, and the subsequent occurrence of developmental dyslexia in school-age children, the study aims to contribute to a better understanding of the origins of dyslexia, thereby enriching the existing knowledge base. This paper outlines the intended approach for the abovementioned systematic review.

## Methods

### Developed protocol

We undertook comprehensive searching following the reporting guidelines outlined in the Preferred Reporting Items for Systematic Review and Meta-analysis Protocols [36]. Our protocol has been registered with the International Prospective Register of Systematic Reviews (PROSPERO) database (registration number: *blinded for review*). The original trial protocol was published elsewhere (*blinded for review*) and is available in S1 File. This report followed set of items for reporting in systematic reviews according to the PRISMA Statement [37]. The PRISMA checklist is shown in S2 File. The review question was as follows: Is prenatal hypoxia associated with developmental learning disorder in school-age children?

### Eligibility criteria

Studies were selected according to pre-specified eligibility criteria following the PICOS model presented in Table 1. The search was not restricted to articles on research conducted in the location of this study.

**Table 1:** Eligibility criteria.

Eligibility criterion	Description
Population (types of participants)	Participants of primary school age diagnosed with DL according to national standards. Children born prematurely are excluded (delivery up to 36,9 weeks of gestation). No restrictions on sex or nationality.
Exposure of interest (independent variable)	Studies with evidence of prenatal/perinatal hypoxia indicated in the child’s medical records.
Comparator/Control	Participants of primary school age without a history of prenatal/perinatal hypoxia. No restrictions on nationality.
Outcomes (dependent variable)	Identification of association between prenatal hypoxia and developmental learning disorder with impairment in reading.
Study type	Inclusion of observational studies comprising cohort and case-control designs.

### Search strategy

#### Information sources

We searched the National Medical Library, PsycINFO, Web of Science, EMBASE, DARE, and the Cochrane Library. In addition to exploring electronic databases, we conducted manual searches for relevant data. The search encompassed all publication years, sample sizes, and languages (with the condition that an English abstract translation was accessible). We eliminated editorials, letters, theses, conference reports, systematic reviews, and theoretical articles from consideration.

#### Search

Medical Subject Headings [38] or equivalent as well as text word terms and words related to the nosological unit were used to implement the literature search strategies. Boolean operators and proximity operators (parentheses and quotations) were also used. The search strategy included terms relating to the outcome and exposure of interest, and was presented as follows: (Learning Development Disorders [MeSH] OR Learning Disabilities OR dyslex* OR legasthenia OR learning disorder OR learning difficult* OR learning disabilit* OR LD OR RD OR SLD OR LRD OR DLD OR reading difficult* OR reading disabilit* OR reading impairment OR reading disorder* OR impairment in reading) and (hypoxia OR asphyxia OR oxygen deficiency OR oxygen shortage OR asphyxiation OR suffocation).

The initial search strategy (comprising search terms and filters) was tested on PubMed in April 2023 to assess its capability in identifying potentially relevant articles. We examined 10 articles in this manner. This preliminary test enabled us to enhance the search criteria.

### Study selection

One of the reviewers conducted database searches and manually examined the reference lists of already included articles. Any potentially relevant articles were imported into the EndNote reference management software [39], and duplicates were identified and eliminated. Titles and abstracts were independently reviewed by two authors to determine their eligibility based on the pre-defined criteria. Reasons for rejections were documented, and disagreements were resolved through discussions with another reviewer.

To ensure the effectiveness of the eligibility criteria, we pre-tested a sample subset of reports. The eligibility criteria for each study were assessed in a prioritised order, beginning with participants and proceeding to exposure of interest, the comparator, outcome, and study design. In this approach, the primary reason for exclusion was the first "no" response, and subsequent criteria were not evaluated. The reasons for exclusion were recorded.

During these assessments, five articles were excluded because of the absence of a comparison group. Both reviewers reached consensus in their assessments.

### Data extraction and management

Two authors independently extracted data from the studies using a purpose-designed pre-piloted data extraction form. Experts in the field of clinical psychology with a solid understanding of learning disorders performed this task. These experts received training in entry coding. Any inconsistencies were resolved through consultation and discussion with another reviewer. In cases where discrepancies could not be resolved internally, the study authors were contacted via email. If contacting the authors proved unsuccessful, any discrepancies were noted in the review.

Subsequently, the collected data were amalgamated across multiple data collection forms.

The following information was extracted from the studies:

The study’s characteristics encompassed:

- Information about the studies, including the first author’s name, publication date, DOI number, and country where the study was conducted
- The study design, differentiating between cohort and case-control designs

Regarding respondents, the following characteristics were examined:

- Demographic details like age and sex
- Language factors such as native language and whether they were monolingual or bilingual
- Educational background and level of intelligence

Regarding developmental learning disorders, we focused on:

- The type of reading difficulties, differentiating between formal diagnoses and poor readers
- Subtypes of developmental learning disorders, such as dyslexia, dysorthography, and reading or math problems
- The criteria used for diagnosis and whether they were based on DSM, ICD, or national standards
- The professionals responsible for the diagnosis (assessors, psychologists, psychiatrists, or others)
- The presence of any comorbidities

Characteristics related to hypoxia included:

- The source of information about hypoxia: was it from medical records or parental accounts?
- The causes of hypoxia
- Methods employed for hypoxia treatment
- Labour type and APGAR scores

The study’s results included:

- Effect sizes indicating the relationship between hypoxia and dyslexia, which might include Pearson’s r for correlational analyses or semi-partial correlations/standardised beta coefficients for multivariate analyses
- Quantitative data showcasing the relationship between hypoxia and dyslexia

#### Assessment of risk of bias in included studies

To assess the risk of bias in included studies will use the Newcastle—Ottawa Scale (NOS) [40] with two independent versions for cohort and case-control studies. We considered three domains including selection, comparability, and in respect to the study type—outcome (cohort studies) or exposure (case-control studies). Details could be find the protocol (S1 File). We didn’t plan to evaluate an overall quality of evidence since there are no plans for a meta-analysis.

#### Strategy for data synthesis

Synthesis of the data collected from the included studies are presented in table and narrative form following the Synthesis Without Meta-analysis (SwiM) guidelines [41]. We did not plan to conduct a meta-analysis, because we expected high heterogeneity of the data measurement tools. Details have been published elsewhere (*blinded for review*).

## Results

### Results of the search

We performed the database searches on 1 April 2023, and identified 1,336 records in the National Medical Library (41), PsycINFO (50), Web of Science (527), EMBASE (714), and DARE (4). No studies were identified after searching the references lists of these records. Furthermore, 401 duplicates were found, including 241 automatically detected by EndNote and 160 manually removed. After excluding duplicates and clearly irrelevant papers, 3 pairs of reviewers examined the full-text articles of 11 potentially relevant studies.

The evaluating teams (pairs) received 309 items (team A), of which evaluator-1 included 1 item and evaluator-2 included 0 items. This discrepancy was assessed positively by reviewer-1, and the paper was included in further analyses. Out of the 309 items received by Team B, evaluator-1 included 7 items, and evaluator-2 also included 7 items. The assessments were consistent for 5 records, and 2 discrepancies were assessed positively by reviewer-2. Therefore, 7 papers were included in further analyses. Team C received 317 items, of which evaluator-1 included 4 items and evaluator-2 included 40 items. Furthermore, 36 discrepancies were found, of which 1 was assessed positively by reviewer-3. Thus, 1 paper was included in further analyses. As such, 9 papers were included in the next stage of evaluation. For one article (from 1987), neither the abstract nor full text could be found. Due to the lack of information on the corresponding author, the publishing house was contacted directly with a request to make the article available. The request to the publishing house was sent twice (20 July 2023 and 8 August 2023) without a response. At this stage, 4 more articles were rejected for the following reasons: 3 articles did not meet the "article type" criteria (1 thesis, 1 systematic review, 1 theoretical), and 1 was a duplicate. Two authors then independently screened the remaining 5 full reports, and decided whether they met the inclusion criteria and provided reasons in cases of rejection. Clinical psychology researchers with expertise in the area assessed the relevance of the studies.

**Fig 1:**
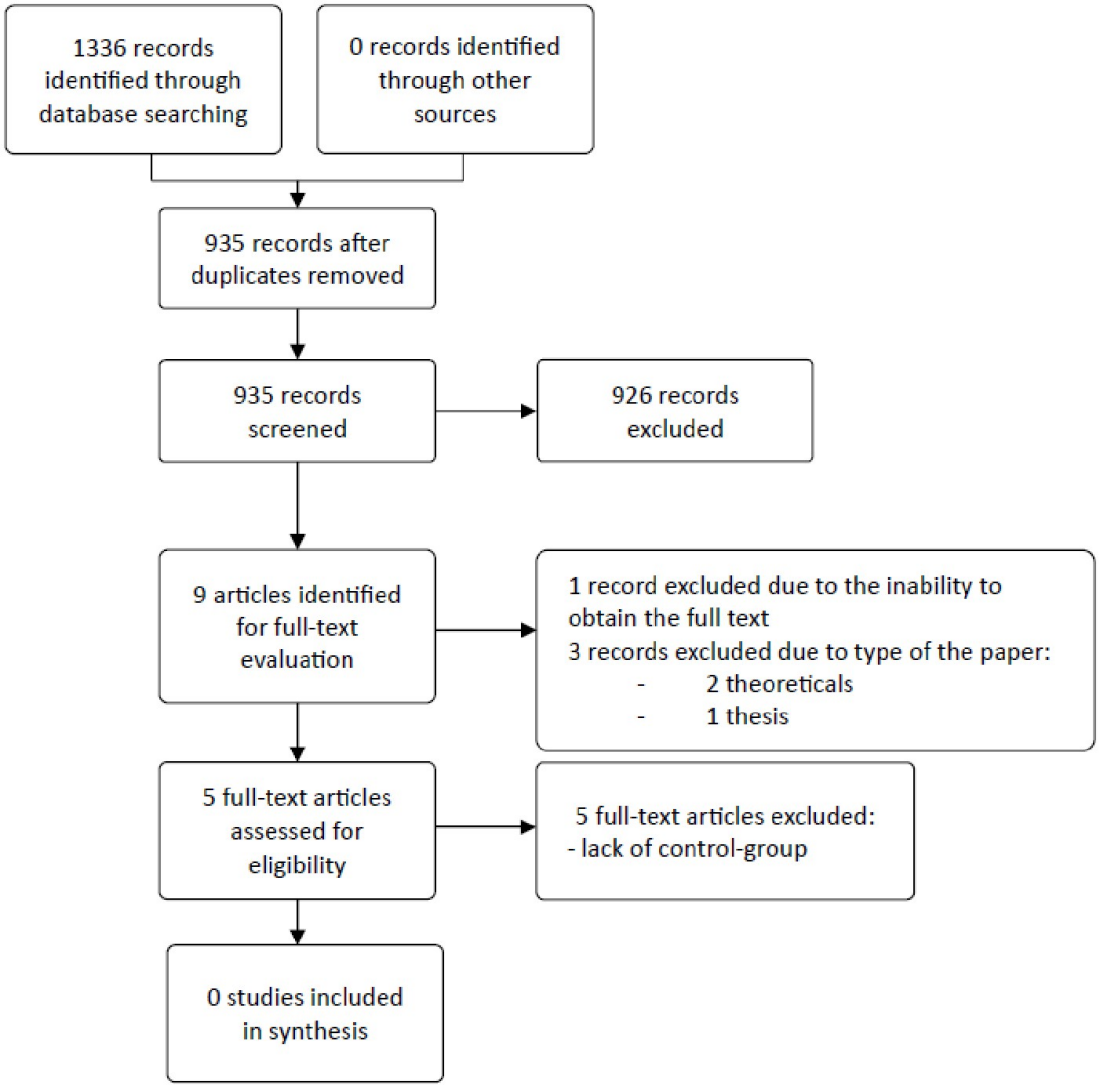
Study flow diagram.

Finally, no articles met the pre-specified eligibility criteria. Fig 1 shows the selection process.

The excluded studies are listed in Table 2. The primary reasons for exclusion of all these articles was lack of a control group. Additionally, other exclusion criteria include: for 3 articles (no. 1, 4, 5; see Table 2) lack of a formal dyslexia diagnosis, for 1 article (no. 2, see Table 2), the epidemiological nature of the study, and for 1 article (no. 3, see Table 2), lack of a group of children with dyslexia and hypoxia.

**Table 2:** List of studies excluded studies in the eligibility stage.

Item no	Full reference
1.	Gatten, S. L., Arceneaux, J. M., Dean, R. S., & Anderson, J. L. (1994). Perinatal risk factors as predictors of developmental functioning. *International Journal of Neuroscience*, *75*(3–4), 167–174. https://doi.org/10.3109/00207459408986300 [42]
2.	Liu, L., Wang, J., Shao, S., Luo, X., Kong, R., Zhang, X., & Song, R. (2016). Descriptive epidemiology of prenatal and perinatal risk factors in a Chinese population with reading disorder. *Scientific Reports*, *6*, 36697. https://doi.org/10.1038/srep36697 [15]
3.	Njiokiktjien, C., de Sonneville, L., & Vaal, J. (1994). Callosal size in children with learning disabilities. *Behavioural Brain Research*, *64*(1–2), 213–218. https://doi.org/10.1016/0166-4328(94)90133-3 [43]
4.	Philip S. S. (2017). Setting up of a cerebral visual impairment clinic for children: Challenges and future developments. *Indian Journal of Ophthalmology*, *65*, 30–34. https://doi.org/10.4103/0301-4738.202303 [44]
5.	Rodríguez, B. H. M. C. (2013). Learning difficulty in 10 years of monitoring in relation to prenatal and perinatal history of high-risk newborns. *Revista Mexicana de Neurociencia*, *14*(5), 249–253. [45]

### Risk of bias

No eligible studies fulfilled the eligibility criteria of this systematic review.

## Discussion

The aim of this systematic review was to evaluate the association between hypoxia and dyslexia by assessing the relationship between a health-related event at the stage of labour and the occurrence of an outcome among children of school age. Despite the extensive search method employed, we did not find any published studies on the subject matter. As there were no eligible studies, the association between hypoxia and developmental dyslexia remains unknown.

We were surprised to find that no studies meeting the established eligibility criteria could be located. In clinical practice, a connection is often indicated between perinatal abnormalities (including oxygen deprivation) and dyslexia. In addition, as dyslexia is a neurodevelopmental disorder and oxygen deprivation significantly influences nervous system development, the absence of research investigating the link between hypoxia and dyslexia appears to be a substantial gap.

The review conducted allowed the identification of several studies suggesting a correlation between perinatal hypoxia and dyslexia (or broader learning disorders). However, due to their epidemiological nature, lack of a control group, and absence of a formal diagnosis of dyslexia, these studies were not included in the systematic review. Nevertheless, the descriptive epidemiological study, conducted by Liu et al. [15], may provide valuable insight into the relationship between hypoxia and dyslexia. This research indicates that perinatal oxygen deprivation is a significant risk factor for dyslexia. Among the various perinatal abnormalities analysed (e.g. difficult vaginal delivery, birth injury, neonatal aspiration pneumonia, neonatal jaundice, neonatal infectious diseases), hypoxia most distinctly differentiated dyslexics from non-dyslexics. In total, 34,748 Chinese students (grades 3–6) were included in the study, among whom 1200 students with dyslexia were identified. Respectively, 3.76% of this group experienced neonatal asphyxia. In the non-dyslexic group, 1.42% of students experienced neonatal asphyxia. This implies that the occurrence of asphyxia is over 2.5 times higher in the group of students with dyslexia. A limitation of this study is its retrospective nature and the potential bias associated with the recall of outcomes.

In certain epidemiological studies where perinatal hypoxia is recorded, students with dyslexia are included in heterogeneous groups of children with neurodevelopmental disorders [45] or under the broader category of "learning disorders". Both these [45] prevent a precise determination of the risk factor’s relationship solely to dyslexia. Nevertheless, in all these studies, it emerges as a potentially significant risk indicator for this disorder. Hence, the conducted review provides a basis for further in-depth investigation into the role of perinatal hypoxia in shaping the risk of dyslexia through clinical control studies.

Research examining physiological variables like perinatal factors and their impact on the subsequent cognitive abilities of students tends to utilise broad qualitative categories such as learning disabilities rather than focusing on specific neurodevelopmental disorders like intellectual disability, dyslexia, or dyscalculia. Psychological research in this area often encounters a challenge in accessing medical records, which acts as a barrier. As a result, psychologists frequently rely on indirect methods like interviews to gather data. Therefore, it seems important that research on the relationship between perinatal burdens and learning disorders in the years of school education be conducted by interdisciplinary teams of specialists, including physicians, psychologists, and epidemiologists.

The existing studies that explore the presence of perinatal hypoxia in the developmental history of students with dyslexia primarily adopt an epidemiological approach. However, a gap exists in terms of clinical and case-control studies within this domain.

The lack of eligible studies might be due to the possibility of publication bias. Even in this study, we applied a comprehensive searching strategy for both an electronic and a manual search and did not include grey literature. However, data produced outside of traditional publishing including master and PhD theses, book chapters, books, reports, policy literature, government documents, speeches, and so on might be an important source of evidence. Therefore, it is important that the searching strategy consider non-published research findings. The lack of eligible records may also be because of restrictive inclusion criteria in terms of study design. We included only case-control and cohort studies, such as studies that included a control group.

Therefore, as asphyxia is a highly complex phenomenon, future research should also consider factors that characterize it, such as the timing of the insult or the distinctions between chronicity and acuity, among others, rather than solely focusing on its presence. This is crucial because these factors may significantly influence the observed outcomes [18].

Systematic reviews without results are called "empty reviews" and are prevalent in the literature. Yaffe et al. [46] reported that approximately 1 in 10 Cochrane reviews did not include results. Considering that no articles met the inclusion criteria, the current systematic review shows gaps in knowledge about the association between a health-related event—hypoxia during pregnancy or delivery and outcomes—and developmental dyslexia in school-age children at a current point in time. Due to the restriction of eligibility criteria, it might also be relevant to undertake a scoping review rather than systematic review. The protocol of the current systematic review was pre-registered with PROSPERO to minimise reporting bias. By pre-registering the protocol, readers could notice that the results of the systematic review do not present post-hoc amendments to the review methodology. We hope that the lack of evidence in this subject area will provide a basis for colleagues pursuing future research on this topic.

## Conclusions

After a systematic search of the literature, we found no records that consider as an exposure the presence of hypoxia during pregnancy or delivery and consider as an outcome dyslexia in school-age children as defined by the ICD-11 classification of neurodevelopmental disorders. Thus, the association between a health-related event—hypoxia during pregnancy or delivery and outcomes—and developmental dyslexia in school-age children remains unknown in the developmental dyslexia aetiology.

## Supporting information

S1 File Developmental learning disorders in children with prenatal/perinatal exposure to hypoxia: A systematic review protocol. (PDF)

S2 File PRISMA checklist. (PDF)

## Abbreviations

CPR: cardiopulmonary resuscitation
DLD: Developmental Learning Disorder
DSM-5: The *Diagnostic* and Statistical Manual of Mental Disorders, Fifth Edition
HIE: Hypoxic-Ischemic Encephalopathy
ICD-11: International Classification of Diseases 11th Revision
LD: Learning Disabilities
NOS: Newcastle–Ottawa Scale
PICOS: Population, Intervention, Comparator (Control), Outcome, Study Design (Setting)
PRISMA-P: Preferred Reporting Items for Systematic Review and Meta-analysis Protocols
PROSPERO: International Prospective Register of Systematic Reviews
RD: reading difficulties
SLD: Specific Learning Disability
SwiM: Synthesis Without Meta-analysis (SwiM)
