# Supplementary material for: Developmental dyslexia in children with perinatal exposure to hypoxia: A systematic review
Source: PLoS One. 2024 Sep 12;19(9):e0308497. doi: 10.1371/journal.pone.0308497 (PMC11392324; doi:10.1371/journal.pone.0308497)
Supplement: S1 File — (PDF) [file pone.0308497.s001.pdf]

PLOS ONE

STUDY PROTOCOL

Developmental learning disorders in children with prenatal/perinatal exposure to hypoxia: A systematic review protocol

Bartosz M. Radtke[1]*, **Urszula Sajewicz-Radtke**[1], **Łucja Bieleninik**[2,3], **Małgorzata Lipowska**[2]

1 Laboratory of Psychological and Educational Tests, Gdańsk, Poland, **2** Institute of Psychology, University of Gdansk, Gdańsk, Poland, **3** GAMUT—The Grieg Academy Music Therapy Research Centre, NORCE Norwegian Research Centre, Bergen, Norway

* radtke@pracowniatestow.pl

Abstract

Background

Developmental learning disorder (DLD) belongs to neurodevelopmental disorders because it results from the developmental neurodiversity of the brain. The main causes of DLD are genetics, but environmental factors, like inadequate supply of oxygen during pregnancy or labor, are considered.

Methods

Our search strategy will consist of electronic databases (PubMed, PsycINFO, Web of Science, EMBASE, and Cochrane Library) and hand searching. The observational studies including cohort and case-control studies will be included. The primary outcome will be (DLD). Screening and eligibility will be done independently by two reviewers based on pre-specified eligibility criteria. Data extraction will be based on a pre-pilot data extraction form, and conducted by two authors independently. Study quality will be assessed by two authors independently. Any discrepancies identified at any stage of the review will be resolved by discussion or/and consultation with another reviewer. We plan a narrative and tabular summary of the findings.

Discussion

This systematic review of aetiology follows the traditional approach to evidence-based healthcare. This secondary research will assess the association between hypoxia and DLD by assessing the relationship of health-related event and outcome and examining the association between them. This review can provide information for healthcare professionals and policymakers indicating whether taking into account information about hypoxia should be permanently included in the diagnostic ontogenetic interview in the process of diagnosing neurodevelopmental disorders.

Check for updates

OPEN ACCESS

Citation: Radtke BM, Sajewicz-Radtke U, Bieleninik Ł, Lipowska M (2023) Developmental learning disorders in children with prenatal/perinatal exposure to hypoxia: A systematic review protocol. PLoS ONE 18(10): e0293067. https://doi.org/10.1371/journal.pone.0293067

Editor: Stefan Grosek, University Medical Centre Ljubljana (UMCL) / Faculty of Medicine, University Ljubljana (FM,UL), SLOVENIA

Received: July 22, 2023

Accepted: October 4, 2023

Published: October 20, 2023

Copyright: © 2023 Radtke et al. This is an open access article distributed under the terms of the Creative Commons Attribution License, which permits unrestricted use, distribution, and reproduction in any medium, provided the original author and source are credited.

Data Availability Statement: No datasets were generated or analysed during the current study. All relevant data from this study will be made available upon study completion.

Funding: This project is funded by statutory funds from the Laboratory of Psychological and Educational Tests in Gdańsk. The funding body does not influence the design of the study and the writing of the manuscript. The funders did not and will not have a role in study design, data collection

and analysis, decision to publish, or preparation of the manuscript.

Competing interests: The authors have declared that no competing interests exist.

Abbreviations: DLD, Developmental Learning Disorder; DSM, Diagnostic and Statistical Manual of Mental Disorders; HIE, Hypoxic–ischemic Encephalopathy; ICD, International Statistical Classification of Diseases and Related Health Problems; MeSH, Medical Subject Headings; NOS, The Newcastle–Ottawa Scale; NS, Narrative Synthesis; PRISMA, Preferred Reporting Items for Systematic Reviews and Meta-Analysis; PROSPERO, Prospective Register of Systematic Reviews.

Systematic review registration

PROSPERO: CRD42022371387.

Background

Developmental learning disorder (DLD) is an umbrella term for developmental dyslexia, as well as difficulties in writing and arithmetic, introduced by the World Health Organisation (WHO) in the latest International Statistical Classification of Diseases and Related Health Problems (ICD–11) [1]. Developmental dyslexia is a neurodevelopmental disorder diagnosed in children with normal intelligence and sensory abilities who exhibit reading difficulties with accurate and/or fluent word recognition, spelling, and decoding abilities across languages. Dyslexia is classified as a neurodevelopmental disorder because it results from the developmental neurodiversity of the brain, which is a biological deviation from the typical neurodevelopmental trajectory [2]. We already know that one of the main causes of dyslexia is genetics [3–5], but environmental factors are an interesting area of research into the pathomechanism of dyslexia, in particular inadequate supply of oxygen during pregnancy or labor [6]. Hypoxia (defined as body tissues being inadequately oxygenated) and related conditions constitute the majority of perinatal injuries [7]. Hypoxic–ischemic encephalopathy (HIE) is a brain injury caused by oxygen deprivation due to hypoxic or anoxic injuries. Hypoxia involves reduced blood oxygenation to the brain. Ischemia involves the diminished blood flow to the brain. HIE occurs in 1 to 8 per 1000 live full-term births [8] and 25% of affected newborns have develop severe and permanent neuropsychological sequelae [9], including intellectual disability, visual motor or visual perceptive dysfunction, increased hyperactivity, cerebral palsy, and epilepsy [10, 11]. Some researchers point out that some patients with HIE may develop learning disabilities [12–14]–in particular, reading difficulties [15–17]. A Chinese study [6] on difficult natural delivery and perinatal hypoxia found that the percentage of dyslexic children in these cohorts was higher than that of non-dyslexic children (3.12% vs. 1.75% and 3.76% vs. 1.42% of the overall sample for difficult natural delivery and perinatal hypoxia, respectively).

Rationale for the review

To the best of our knowledge, there is no systematic review nor meta-analysis on the association between exposure to prenatal/perinatal hypoxia and the occurrence of DLD symptoms at school age. Therefore, our motivation is to conduct the first systematic review of previous published observational studies (i.e., cohort studies, and case-control studies) that consider, as an exposure, presence of hypoxia during pregnancy or delivery and consider, as an outcome, DLD in the school-age children, as defined by the DSM-5 classification of neurodevelopmental outcomes. The understanding of the association between health-related event—hypoxia during pregnancy or delivery and outcome—DLD in school-age children will provide evidence in aetiology of DLD and thus, improve our understanding of the state of knowledge. The aim of this paper is to present the protocol for the aforementioned systematic review.

Methods

We followed the Preferred Reporting Items for Systematic Review Protocols (PRISMA-P) to perform a comprehensive literature search [18]. Our protocol has been registered with the International Prospective Register of Systematic Reviews (PROSPERO) database (registration

number: CRD42022371387). The research objective is to synthesize the current research on the association between hypoxia during pregnancy or delivery and dyslexia in school-age children. The review question is as follows: whether prenatal hypoxia is associated with developmental learning disorder in school age?

Eligibility criteria

Studies will be chosen based on the pre-specified below mentioned eligibility criteria.

Population (types of participants). The participants in the included studies will have been of primary school age, with no restrictions on sex or nationality, and have been diagnosed with DLD. DLD should have been diagnosed according to the national standards. Due to the research question of aetiology, we aim to exclude prematurely born children (defined as delivery up to 36;9 weeks of gestation).

Exposure of interest (independent variable). The review will include studies that consider, as an exposure, presence of prenatal/perinatal hypoxia indicated in the child's medical records.

Comparator/control. We will include studies with participants of primary-school age, with no restrictions on nationality and without history of prenatal/perinatal hypoxia.

Outcome (dependent variable). The review will include studies that consider, as an outcome, presence of developmental learning disorder.

Study Type. We will include observational studies including cohort and case-control studies.

Location. We will not impose any restrictions on the location of the conducting of the research.

Search strategy

Information sources. We will search the National Medical Library, PsycINFO, Web of Science, EMBASE, DARE, and the Cochrane Library. Electronic database searching will be complemented by manual data searches. Reference lists of the included review articles will be checked to identify any additional studies. The search will be not restricted to any year of publication, sample size, or language (provided an English language translation of the abstract is available). We will exclude editorials and letters.

Search. Medical Subject Headings [19] or equivalent and text word terms and words related to the nosological unit will be used to develop literature search strategies. Boolean operators and proximity operators (parentheses and quotations) will also be used. The search strategy will include terms relating to outcome (Learning Development Disorders [MeSH] OR Learning Disabilities OR dyslex* OR legasthenia OR learning disorder OR learning difficult* OR learning disabilit* OR LD OR RD OR SLD OR LRD OR DLD OR reading difficult* OR reading disabilit* OR reading impairment OR reading disorder* OR impairment in reading) and exposure of interest (hypoxia OR asphyxia OR oxygen deficiency OR oxygen shortage OR asphyxiation OR suffocation). The initial search strategy (including searching terms and filters) has already been piloted on PubMed in June 2023 to investigate whether it can find potentially relevant reports.

Study selection

One of the reviewers will search databases as well as manually search the reference lists of already-included articles. Any articles that may be relevant will be extracted to EndNote reference management software [20] and duplicates will be identified and removed. Titles and abstracts will be screened by two authors independently for eligibility for inclusion under the

above-defined criteria and will give reasons for any rejections. Discrepancies at this stage will be resolved through discussion with another reviewer. We will pre-test the eligibility criteria on a sufficient sample of reports. The full texts of reports will be acquired for all titles that appear to meet the inclusion criteria or in cases where there is any uncertainty. The full reports will then be screened by two authors independently, who will decide whether they meet the inclusion criteria and provide reasons in cases of rejection. Clinical psychology researchers with expertise in the area will assess the relevance of the studies. The corresponding authors will be asked to provide additional information whenever necessary to resolve questions of eligibility. We will assess the eligibility criteria for each study in order of importance, starting with the population, followed by exposure of interest, comparator, outcome, and study design. In this strategy, the first 'no' response constitutes the primary reason for exclusion from the study and the remaining criteria will not be assessed. Reasons for exclusion will be recorded. A formal measure of agreement will be used to describe the extent to which assessments by two authors are the same. Another reviewer will resolve disagreements at the assessment eligibility stage. In cases where the same study is described in multiple reports, they will be merged based on the matching of author names, numbers of participants and baseline data, location and setting, and duration of the study. If any uncertainty remains, we will contact the corresponding authors. The review authors will not be blind to the journal titles, study authors, or their institutions. A flow chart based on the PRISMA template [21] will be created, showing details of studies included and excluded at each stage of the study selection process.

Data extraction and management

Data will be extracted from the studies independently by two authors based on a purpose-designed pre-piloted data extraction form. This will be done by content area experts in the field of clinical psychology who are familiar with DLD and who will receive training in the coding of entries. Any discrepancies will be resolved by consultation/discussion with another reviewer. If a discrepancy cannot be resolved, we will contact by e-mail the study authors; if we are unsuccessful in doing so, we will report the discrepancies in the review. We will attempt to obtain any missing data from corresponding authors by e-mail. For different reports describing the same study/project, data will be extracted from each report separately and combined across multiple data collection forms afterwards.

The following information will be extracted from the studies:

- Characteristics of studies:
- first author, date of publication, DOI number, country of the study;
- study design (cohort vs. case-control).
- Characteristics of the respondents:
- age, sex;
- native language, languages spoken (mono vs. bilingualism); and
- years of education, intelligence level.
- Characteristics of Developmental Learning Disorder:
- type of DLD (formal diagnosis vs. poor readers);
- DLD subtype (dyslexia, dysorthography, reading and math problems);
- criteria of diagnosis (DSM, ICD, national standards);

- diagnosis provider (assessor, psychologist, psychiatrist, other), and
- comorbidities.
- Hypoxia characteristics:
- source of information about hypoxia (medical record vs. parental information);
- cause of hypoxia;
- methods of hypoxia treatment; and
- type of labor, APGAR score.
- Results
- effect sizes for the relation between hypoxia and dyslexia (e.g. Pearson's r for correlational analyses; semi-partial correlations or standardized beta coefficients for multivariate analyses)
- quantitative data of relation between hypoxia and dyslexia

Assessment of risk of bias in included studies. The Newcastle–Ottawa Scale (NOS) will be used to assess the risk of bias in each included study [22]. Two independent versions of NOS will be used to evaluate cohort and case-control studies. The NOS judge eight items, categorized into three domains including selection, comparability, and in respect to the study type —outcome (cohort studies) or exposure (case-control studies). Each study could receive a maximum of nine stars (one star for each numbered item within the selection and outcome categories, and a maximum of two stars for comparability) [23]. Two review authors will independently assess potential biases while any discrepancies will be resolved by discussion between reviewers; if necessary, content area experts from clinical trial methodology will be included in the discussion. Risk of bias will be categorized as "good", "fair", and "poor" for converting NOS assessments following guidelines of the Agency for Healthcare Research and Quality (AHRQ). Studies scored for 3 or 4 stars in the selection domain, 1 or 2 stars in the comparability domain, and 2 or 3 stars in the outcome/exposure domain will be considered to represent good quality. Studies rated 2 stars in the selection domain, 1 or 2 stars in the comparability domain, and 2 or 3 stars in the outcome/exposure domain were considered to represent fair quality. Papers evaluated as being 0 or 1 stars in the selection domain, 0 stars in the comparability domain, and 0 or 1 stars in the outcome/exposure domain were considered to be poor quality [24]. We don't plan to evaluate an overall quality of evidence since there are no plans for a meta-analysis.

Strategy for data synthesis. Both narrative and tabular summary will be used to present evidence of the relationship of health-related event and outcome and examining the association between them. We do not plan to conduct a meta-analysis because we expect high heterogeneity of data measurement tools across country.

The Narrative Synthesis (NS) guidelines will allow us to transparently report the data from the included studies [25]. We will perform data synthesis for each outcome of DLD subtype (dyslexia, dysorthography, reading and math problems) separately. In the event of constructs overlapping, they will be combined and discussed together as DLD.

Narrative synthesis will be done following domains:

1. Developing a preliminary synthesis of findings of included studies
2. Exploring relationships within and between studies

a. Developing a theory explaining the role of hypoxia as a aetiological risk factor for neurodevelopmental disorders in the form of DLD depending on the DLD subtype (dyslexia, dysorthography, reading and math problems);

b. Assessing if the presence of hypoxia in a group of children with DLD increases the risk of comorbidity of other neurodevelopmental disorders.

c. Assessing if the occurrence of perinatal hypoxia increases the risk of DLD and DLD with comorbid neurodevelopmental disorders depending on the sex of the child.

3. Assessing the robustness of the synthesis

Additionally, if it is possible to collect sufficient information, we will analyze the following subgroups:

- children with a history of prenatal/perinatal hypoxia caused by various factors;
- children with different types of DLD;
- children with different levels of severity of DLD.

Discussion

The proposed systematic review will contribute to healthcare and psychology filed by providing evidence for clinicians and policymakers on the burden of disease–in particular, in the field of diagnosing learning disorders and early recognition of risk of these disorders. The questions of association typically address etiology of DLD remain unsolved. Evaluating the association between hypoxia and DLD by assessing the relationship of health-related event at the stage of labor and the occurrence of outcome at the school age will support the need for detailed recording of all physiological indicators that may be helpful in predicting the risk of possible learning disorders. Diagnosing of DLD is not a "here and now" diagnosis, but an assessment of the entire developmental trajectory [26]. Therefore, in the process of diagnosing DLD, we must refer to the individual's developmental history from birth. The cooperation of healthcare practitioners and developmental psychologists will allow the developing of a very early warning system for the risk of DLD and early preventive and therapeutic intervention. Early intervention is key to minimizing the likelihood of the presence of a learning disorder during school years [27]. Confirmation of the relationship between hypoxia and DLD would be a strong basis for including questions about this aspect of the delivery and the condition of the newborn into standard psychological interviews used when diagnosing it. The inclusion of this evidence-based indicator would enhance the accuracy of the diagnosis of DLD, which is not an easy process due to its complex pathomechanism [28–30].

Efforts will be undertaken by the authors to disseminate the findings of this systematic review across a broad spectrum of healthcare practitioners and policy makers at the national level. This dissemination will primarily occur through the distribution of a newsletter that serves as a conduit for information exchange among key stakeholders, including directors and clinicians affiliated with diagnostic centers within the education and healthcare sectors.

Furthermore, at the international level, the research outcomes will be made accessible through inclusion in prominent data repositories designed to amalgamate information from diverse sources (among others: Systematic Review Data Repository™).

Supporting information

S1 Checklist. PRISMA-P (Preferred Reporting Items for Systematic review and Meta-Analysis Protocols) 2015 checklist: Recommended items to address in a systematic review

protocol*.
(PDF)

Author Contributions

Conceptualization: Bartosz M. Radtke, Urszula Sajewicz-Radtke, Łucja Bieleninik, Małgorzata Lipowska.

Data curation: Bartosz M. Radtke, Urszula Sajewicz-Radtke, Łucja Bieleninik, Małgorzata Lipowska.

Funding acquisition: Bartosz M. Radtke.

Project administration: Bartosz M. Radtke.

Supervision: Łucja Bieleninik, Małgorzata Lipowska.

Writing – original draft: Bartosz M. Radtke, Urszula Sajewicz-Radtke, Łucja Bieleninik, Małgorzata Lipowska.

Writing – review & editing: Bartosz M. Radtke, Urszula Sajewicz-Radtke, Łucja Bieleninik, Małgorzata Lipowska.

References

1. WHO. International classification of diseases for mortality and morbidity statistics (11th Revision): Retrieved from https://icd.who.int/browse11/l-m/en; 2019.
2. Norbury CF, Sparks A. Difference or disorder? Cultural issues in understanding neurodevelopmental disorders. Developmental Psychology. 2013; 49(1):45–58. https://doi.org/10.1037/a0027446 PMID: 22390668
3. Grigorenko EL. Developmental dyslexia: an update on genes, brains, and environments. Journal of Child Psychology and Psychiatry. 2001; 42(1):91–125. PMID: 11205626
4. Mascheretti S, De Luca A, Trezzi V, Peruzzo D, Nordio A, Marino C, et al. Neurogenetics of developmental dyslexia: from genes to behavior through brain neuroimaging and cognitive and sensorial mechanisms. Translational Psychiatry. 2017; 7(1):e987. https://doi.org/10.1038/tp.2016.240 PMID: 28045463
5. Erbeli F, Rice M, Paracchini S. Insights into Dyslexia Genetics Research from the Last Two Decades. Brain Sciences. 2022; 12(1):27.
6. Liu L, Wang J, Shao S, Luo X, Kong R, Zhang X, et al. Descriptive epidemiology of prenatal and perinatal risk factors in a Chinese population with reading disorder. Scientific Reports. 2016; 6(1):36697.
7. Martin RP, Dombrowski SC. Pre- and Perinatal Anoxia and Hypoxia. Prenatal Exposures: Psychological and Educational Consequences for Children. Boston, MA: Springer US; 2008. p. 47–54.
8. Lee BL, Glass HC. Cognitive outcomes in late childhood and adolescence of neonatal hypoxic-ischemic encephalopathy. Clinical and Experimental Pediatrics. 2021; 64(12):608–18. https://doi.org/10.3345/cep.2021.00164 PMID: 34044480
9. Odd D, Heep A, Luyt K, Draycott T. Hypoxic-ischemic brain injury: Planned delivery before intrapartum events. Journal of neonatal-perinatal medicine. 2017; 10(4):347–53. https://doi.org/10.3233/NPM-16152 PMID: 29286930
10. Kurinczuk JJ, White-Koning M, Badawi N. Epidemiology of neonatal encephalopathy and hypoxic–ischaemic encephalopathy. Early Human Development. 2010; 86(6):329–38. https://doi.org/10.1016/j.earlhumdev.2010.05.010 PMID: 20554402
11. Vannucci RC, Perlman JM. Interventions for Perinatal Hypoxic–Ischemic Encephalopathy. Pediatrics. 1997; 100(6):1004–114. https://doi.org/10.1542/peds.100.6.1004 PMID: 9374573
12. van Handel M, Swaab H, de Vries LS, Jongmans MJ. Long-term cognitive and behavioral consequences of neonatal encephalopathy following perinatal asphyxia: a review. European journal of pediatrics. 2007; 166(7):645–54. https://doi.org/10.1007/s00431-007-0437-8 PMID: 17426984
13. Lindström K, Lagerroos P, Gillberg C, Fernell E. Teenage Outcome After Being Born at Term With Moderate Neonatal Encephalopathy. Pediatric Neurology. 2006; 35(4):268–74. https://doi.org/10.1016/j.pediatrneurol.2006.05.003 PMID: 16996401

14. Marlow N, Rose AS, Rands CE, Draper ES. Neuropsychological and educational problems at school age associated with neonatal encephalopathy. Archives of Disease in Childhood—Fetal and Neonatal Edition. 2005; 90(5):F380. https://doi.org/10.1136/adc.2004.067520 PMID: 16113154

15. Becker N, Vasconcelos M, Oliveira V, Santos FCD, Bizarro L, Almeida RMM, et al. Genetic and environmental risk factors for developmental dyslexia in children: systematic review of the last decade. Developmental Neuropsychology. 2017; 42(7–8):423–45. https://doi.org/10.1080/87565641.2017.1374960 PMID: 29068706

16. Cho K, Frijters JC, Zhang H, Miller LL, Gruen JR. Prenatal Exposure to Nicotine and Impaired Reading Performance. Journal of Pediatrics. 2013; 162(4):713–8.e2. https://doi.org/10.1016/j.jpeds.2012.09.041 PMID: 23122624

17. Mascheretti S, Bureau A, Battaglia M, Simone D, Quadrelli E, Croteau J, et al. An assessment of gene-by-environment interactions in developmental dyslexia-related phenotypes. Genes, Brain and Behavior. 2013; 12(1):47–55. https://doi.org/10.1111/gbb.12000 PMID: 23176554

18. Shamseer L, Moher D, Clarke M, Ghersi D, Liberati A, Petticrew M, et al. Preferred reporting items for systematic review and meta-analysis protocols (PRISMA-P) 2015: elaboration and explanation. BMJ: British Medical Journal. 2015; 349:g7647. https://doi.org/10.1136/bmj.g7647 PMID: 25555855

19. Dhammi IK, Kumar S. Medical subject headings (MeSH) terms. Indian journal of orthopaedics. 2014; 48 (5):443–4. https://doi.org/10.4103/0019-5413.139827 PMID: 25298548

20. Bramer W, Bain P. Updating search strategies for systematic reviews using EndNote. Journal of the Medical Library Association: JMLA. 2017; 105(3):285–9. https://doi.org/10.5195/jmla.2017.183 PMID: 28670219

21. Page MJ, McKenzie JE, Bossuyt PM, Boutron I, Hoffmann TC, Mulrow CD, et al. The PRISMA 2020 statement: an updated guideline for reporting systematic reviews. BMJ. 2021; 372:n71. https://doi.org/10.1136/bmj.n71 PMID: 33782057

22. Ettenberger M, Odell-Miller H, Rojas Cárdenas C, Torres Serrano S, Parker M, Camargo Llanos SM. Music Therapy With Premature Infants and Their Caregivers in Colombia–A Mixed Methods Pilot Study Including a Randomized Trial. Voices: A World Forum for Music Therapy, [Sl]. 2014; 14(2).

23. >Wells GA, Wells G, Shea B, Shea B, O'Connell D, Peterson J, et al., editors. The Newcastle-Ottawa Scale (NOS) for Assessing the Quality of Nonrandomised Studies in Meta-Analyses2014.

24. Viswanathan M, Ansari MT, Berkman ND, Chang S, Hartling L, McPheeters M, et al. AHRQ Methods for Effective Health Care Assessing the Risk of Bias of Individual Studies in Systematic Reviews of Health Care Interventions. Methods Guide for Effectiveness and Comparative Effectiveness Reviews. Rockville (MD): Agency for Healthcare Research and Quality (US); 2008.

25. Popay J, Roberts H, Sowden A, Petticrew M, Arai L, Rodgers M, et al. Guidance on the conduct of narrative synthesis in systematic reviews. A product from the ESRC methods programme Version. 2006; 1 (1):b92.

26. Kuppen SEA, Goswami U. Developmental trajectories for children with dyslexia and low IQ poor readers. Developmental Psychology. 2016; 52(5):717–34. https://doi.org/10.1037/a0040207 PMID: 27110928

27. Colenbrander D, Ricketts J, Breadmore HL. Early Identification of Dyslexia: Understanding the Issues. Language, Speech, and Hearing Services in Schools. 2018; 49(4):817–28. https://doi.org/10.1044/2018_LSHSS-DYSLC-18-0007 PMID: 30458543

28. McArthur G, Kohnen S Fau—Larsen L, Larsen L Fau—Jones K, Jones K Fau—Anandakumar T, Anandakumar T Fau—Banales E, Banales E Fau—Castles A, et al. Getting to grips with the heterogeneity of developmental dyslexia. 2013(1464–0627 (Electronic)).

29. Carroll J, Solity J, Shapiro L. Predicting dyslexia using prereading skills: The role of sensorimotor and cognitive abilities. Journal of child psychology and psychiatry, and allied disciplines. 2015; 57. https://doi.org/10.1111/jcpp.12488 PMID: 26662375

30. Pennington B, Santerre-Lemmon L, Rosenberg J, MacDonald B, Boada R, Friend A, et al. Individual Prediction of Dyslexia by Single Versus Multiple Deficit Models. Journal of abnormal psychology. 2012; 121:212–24. https://doi.org/10.1037/a0025823 PMID: 22022952
